# Phenomenal Holism and Cognitive Phenomenology

**DOI:** 10.1007/s10670-021-00501-x

**Published:** 2021-12-09

**Authors:** Martina Fürst

**Affiliations:** Department of Philosophy, University of Graz, Heinrichstrasse 26/5, 8010 Graz, Austria

## Abstract

The cognitive phenomenology debate centers on two questions. (1) What is an apt characterization of the phenomenology of conscious thought? And (2), what role does this phenomenology play? I argue that the answers to the former question bear significantly on the answers to the latter question. In particular, I show that conservatism about cognitive phenomenology is not compatible with the view that phenomenology explains the constitution of conscious thought. I proceed as follows: To begin with, I analyze the phenomenology of our sensory experiences and argue for a *weak phenomenal holism* (WPH) about sensory phenomenology. Next, I explore how WPH can be integrated into the competing accounts of cognitive phenomenology. I argue that, given WPH, conservatism turns out to reduce phenomenal character to a merely concomitant phenomenon that has no explanatory power when it comes to the constitution of conscious thoughts. In contrast, liberalism is explanatorily more powerful in this respect. Finally, I propose a new version of liberalism that explains how phenomenology constitutes conscious thoughts and fits best with WPH.

## Introduction

On a widely shared view, sensory experiences exhibit a phenomenal character. According to the “cognitive phenomenology thesis” (hereinafter: CP-thesis) occurrent cognitive states possess a phenomenal character as well. That means that there is something that it is like to think that I will go hiking tomorrow or to wonder whether the weather will be sunny. The debate about cognitive phenomenology centers on the following question: what is an apt characterization of the phenomenal character of conscious thought?^1^ In particular, is the phenomenal character of conscious thought reducible to the phenomenology of sensory experiences or is it of a sui generis and proprietary kind? To clarify whether the what-it-is-likeness of conscious thought is all sensory is important, since the respective answers shed light on further important questions such as: what role does the phenomenal character of conscious thought play?

In the current literature, we can roughly discern two competing views on the issue. On the “conservative view”^2^ of the CP-thesis the phenomenal character of conscious thought is reducible to sensory phenomenology (Tye & Wright, 2011; Prinz, 2011; Robinson, 2011; Pautz, 2013; Carruthers & Veillet, 2017). What it is like to consciously think that p involves inner imageries, inner speech, motor-sensory responses, emotions etc. Conservatism has the advantage of relying on a fairly uncontroversial kind of phenomenology. However, as I will argue, restricting the phenomenology of thoughts to the sensory comes at the cost of conservatism having little explanatory power. In particular, conservatism cannot explain how phenomenology *constitutes* conscious thoughts.

On the rival view, labeled as “liberalism”, cognitive phenomenology is of a sui generis*, proprietary*, kind (Chudnoff, 2015; Horgan, 2013; Horgan & Tienson, 2002; Kriegel, 2015; Montague, 2016; Pitt, 2004; Siewert, 1998; Strawson, 1994). Liberalism comes in different strengths, depending on whether cognitive phenomenology is seen as irreducible to sensory phenomenology or as modally independent of sensory phenomenology. Hereinafter, I will refer to the first, weaker, view as liberalism+ and to the second view as liberalism*.

Liberalism faces the challenge that its main claim of an additional, sui generis kind of phenomenology is highly controversial. Liberals meet this challenge by providing arguments of various forms to establish the existence of a sui generis, proprietary cognitive phenomenology, the most famous ones among them being the arguments from phenomenal contrast. Arguments from phenomenal contrast take the following form. First, readers are invited to imagine two phenomenally contrasting mental states. Second, it is pointed out that the same sensory-phenomenal elements are involved in these states. Third, it is argued that the phenomenal contrast is best accounted for in terms of a proprietary cognitive phenomenology.

Notably, within the debate about cognitive phenomenology, arguments from phenomenal contrast turn out to be dialectically ineffective. To investigate the reasons for their dialectical ineffectiveness is an interesting task that has received some attention in the literature. For example, Koksvik (2015) argues that *argumentative* uses of the method of phenomenal contrast which aim to entail their target thesis fail because the requirement to provide minimal pairs of the contrast scenarios cannot be met. Moreover, *ostensive* uses of the method of phenomenal contrast, which aim at inducing a first-person experience of proprietary cognitive phenomenology,^3^ fail to convince the conservatives who insist that they cannot find the relevant introspective evidence.^4^ Given the dialectical weaknesses of arguments from phenomenal contrast, new arguments for liberalism are called for which do not suffer from these problems. The goal of this paper is to offer such a new argument for liberalism.

Arguments for liberalism often involve cases in which two subjects allegedly share the *same* sensory phenomenology but have different conscious thoughts. Most phenomenal contrast arguments rely on such cases and describe scenarios that involve, for example, the same auditory imagery but that intuitively give rise to a phenomenal contrast. Well-known examples include the as-of-understanding experience of a particular sentence by a native speaker that phenomenally differs from the puzzlement by a non-native speaker (Horgan, 2013; Horgan & Tienson, 2002; Siewert, 1998; Strawson, 1994) or two different ways of understanding ambiguous sentences (Horgan & Tienson, 2002).^5^ I will approach the debate from a different angle by analyzing how profoundly phenomenology can *diverge* and nonetheless be constitutive of the same conscious thought.

I proceed as follows: I start with isolating the CP-thesis that interests me, namely the thesis that phenomenology is *constitutive* of conscious thought in the sense that the phenomenology *explains* why a target thought is the thought it is. In Sect. 2, I draw attention to the actual phenomenal character of our sensory experiences and introduce the thesis of *weak phenomenal holism* (WPH). Then I explore how WPH can be integrated into the competing accounts of cognitive phenomenology. In Sects. 3 and 4 I argue that, given WPH, conservatism turns out to reduce phenomenal character to a merely concomitant phenomenon that has no explanatory power when it comes to the constitution of conscious thoughts. In Sect. 5, I show that liberalism is explanatorily more powerful in this respect. However, the extant liberal accounts rest on controversial premises. In the final Sect. 6, I develop a variant of liberalism about cognitive phenomenology, the *fusion account,* that explains the phenomenology of thought and fits best with WPH.

## The Explanatory Power of the Cognitive Phenomenology Thesis

The cognitive phenomenology debate not only centers on the question of what an apt characterization of the phenomenology of conscious thought^6^ is, it also focuses on the role this phenomenology plays. What I call the “weak” CP-thesis has it that the phenomenology is merely *accompanying* conscious thought. In contrast, the “strong” CP-thesis states that phenomenology is *constitutive* of conscious thought by constituting (or determining) its content.

At a first sight, the strong and the weak CP-theses seem compatible with both liberalism and conservatism. However, liberalism typically comes with a strong CP-thesis. For example, Pitt (2004, p. 5) holds that proprietary cognitive phenomenology “constitutes its representational content (i.e., is individuative)” and Horgan and Graham (2012) argue that cognitive phenomenology *grounds or determines* thought content.

Conservatism comes in both strengths. In its weak form, conservatism has it that the sensory phenomenology of conscious thought is just a concomitant phenomenon. For example, Robinson tells us: “(T)he particular images I had (when understanding) were not necessary, even for me, for understanding. […] The imagery that accompanies my (eventual) understanding of ((sentence) 1) seems to me to be mere accompaniment, and not the phenomenology of understanding the content.” (2005, p. 550) Weak conservatism is often endorsed with the sole aim of explaining away phenomenal contrast arguments by accounting for the phenomenology of thought in terms of sensory phenomenology (Robinson, 2005, 2011; Tye & Wright, 2011). Since this paper aims at analyzing the *phenomenal constitution* of conscious thought, I will set the weak CP-thesis aside.

Conservatism can also be endorsed as a version of the strong CP-thesis, by holding that sensory phenomenology is *constitutive* of conscious thought. As Bayne and Montague (2011, p. 12) put one such version of conservatism:

(Some theorists) hold that thought always and essentially involves, or is somehow “realized in”, a sensory medium of some sort. […] On this view, a particular thought will be “carried” by a phenomenal state, but the phenomenology of that state will be exclusively sensory.

Notably, there are two ways of fleshing out the claim that sensory phenomenology constitutes conscious thought. On one interpretation, sensory phenomenology *alone* constitutes conscious thought by constituting the thought content. I label this view “strong conservatism”. For example, Prinz (2011, p. 175) can be read in this way, given that he subscribes to concept empiricism and given that thoughts are combinations of concepts.

On another interpretation (e.g., Carruthers & Veillet 2011; Pautz, 2013), sensory phenomenology is only *partly* constitutive of conscious thought in the sense that there would not be a conscious thought without sensory phenomenology. *Some* sensory phenomenology or other is required, but no *particular* sensory phenomenology is needed for a target thought. To fully account for a particular conscious thought, another factor besides phenomenology is needed. I label this view “strong conservatism + ”. The “ + ” indicates that an additional factor is tied to sensory phenomenology to account for the constitution of conscious thought. What distinguishes strong conservatism + from strong conservatism is that the constitution of conscious thought is not explained by sensory phenomenology alone but rather by the conjunction of sensory phenomenology and a further factor. For example, Pautz (2013) defends a strong conservatism + and argues that sensory phenomenology together with functional facts accounts for the constitution of mental content. Since the strong CP-thesis aims to explain how a target thought is the thought it is in terms of the phenomenology, the key question for an analysis of strong conservatism + is the following: does the sensory phenomenology contribute to the *explanation* of why a target thought is the thought it is, or is all the explanatory work done solely by the additional, non-phenomenal factor?

Let me summarize the taxonomy: The label “strong” indicates that phenomenology is *constitutive* of conscious thoughts in the sense that the phenomenology explains why a particular target thought is the thought it is (most plausibly, by determining the thought content). This qualification applies to strong conservatism, strong conservatism + , and to strong liberalism. These are the views I am interested in within this paper. The labels “liberal” and “conservative” point towards a difference in the characterization of the relevant phenomenology; the former allows for a proprietary cognitive phenomenology, whereas the latter grants only sensory phenomenology. Finally, the “ + ” indicates that a further factor, besides the sensory phenomenology, is needed to account for the constitution of conscious thought.

In this paper, I am interested in the strong CP-thesis which aims at explaining the constitution of conscious thought via phenomenology. The reason is that the strong CP-thesis has the potential to explain a wide range of phenomena, such as introspective knowledge about our occurrent thoughts (Pitt, 2011) and, most importantly, the determinacy of mental content (Horgan & Graham, 2012; Pitt, 2004). A strong CP-thesis that explains these puzzles will generate a more sophisticated understanding of conscious thought and thereby deepen our understanding of conscious intentionality in general. In the literature, we find both liberal and conservative views which aim to be explanatorily powerful in this respect. In the following, I investigate which view best explains the phenomenal constitution of conscious thought. But before finding this out, we first have to get clear about the phenomenology of our sensory experiences.

## Weak Phenomenal Holism

Conscious mental states such as seeing the blue sky or hearing the sound of a flute exhibit a specific phenomenal character, a what-it-is-likeness (Nagel, 1974) to be in such states. In the literature on phenomenal consciousness, the examples used often belong to one singular sense modality (e.g., what it is like for Jackson’s Mary (Jackson, 1982) to see colors or for the zombie’s human twin (Block, 1980) to feel pain). These perceptual experiences are then treated as if their phenomenal character could be analyzed in isolation from the other phenomenal properties that are part of the overall phenomenal state of the subject. For some debates, such as the debate about the *hard problem of consciousness* (Chalmers, 1996), this simplification seems harmless. However, within the debate about cognitive phenomenology the situation is different. One question at the center of the debate is whether cognitive phenomenology is reducible to sensory phenomenology. Hence, sensory phenomenology is a key-issue to be clarified. Accordingly, within the debate about cognitive phenomenology the desideratum is to characterize aptly the actual sensory phenomenology we are enjoying.

When it comes to analyzing our actual sensory phenomenology, experimental findings of cross-modal interference show that the phenomenologies of the singular sense modalities are not encapsulated in the sense of being independent of each other.^7^ Rather, the phenomenal character of an experience in one sense-modality differs if the phenomenal character of an experience in another sensory modality varies. For example, visual and auditory experiences interact with each other, as the famous “McGurk effect” (McGurk and MacDonald, 1976) shows. Observers who see a woman mouthing the syllable “ga” and hear an auditory recording of “ba,” report that their auditory experience is of a third phoneme—“da”. That means, the way the visual information is processed is relevant to the character of the auditory phenomenology. The effect is an instance of multisensory integration. Notably, cross-modal interference is not restricted to visual and auditory phenomenology. Tactile experiences interfere with other sense modalities as well. As the “parchment skin illusion” (Jousmäki & Hari, 1998) shows, sound levels can affect tactile sensation: when a person is exposed to an increasing sound, the sound level affects how smooth/rough one’s palms feel when the hands are rubbed together. Moreover, tactile motion-experiences have been found to influence visual experiences and vice versa (Konkle et al., 2009).^8^

The empirical evidence that the sensory-phenomenal parts of an overall phenomenal state or experience^9^ are not independent of each other, as well as recent empirical evidence about multisensory processing—the interaction of processing that originates from different sensory organs—, point towards *phenomenal holism.* If our sense modalities are processed together and interfere with each other in a way that influences the phenomenal parts of the experience, the result is a *holistic* phenomenal character of experience. Phenomenal holism, roughly put, is the view that the phenomenal parts of a total experience^10^ depend on the overall state. Hence, the phenomenal parts are not independent from each other. Phenomenal holism comes in different strengths.

One way to differentiate *strong* holism from *weak* holism is the way the dependence relation between the relevant phenomenal parts is fleshed out. If the dependence is seen as *causal*, the result is weak phenomenal holism. In contrast, if the relation between the relevant phenomenal parts is seen as *constitutive*, the result is a strong phenomenal holism.^11^

Another possibility is to distinguish strong from weak phenomenal holism by considering its scope. Following this line, strong phenomenal holism concerns *all* phenomenal states while weak holism is restricted to a *particular kind* of phenomenal state. Moreover, one might contrast the view that the interdependence applies to *all* parts of the total state with the view that it applies only to *some* of its phenomenal parts.

One example that qualifies as strong phenomenal holism according to both of these distinctions is found in Dainton (2010), who argues that all parts of all experiences are necessarily interdependent. Chudnoff (2015, pp. 128–129) defends a strong phenomenal holism as well, by arguing that all phenomenal states depend for their occurrence on their centrality connections to other phenomenal states. To investigate the kind of dependence and the scope of phenomenal holism in more detail is an important and interesting task.^12^ However, for the present purpose I prefer to stick to the following, minimum, assumption:

WPH: The sensory-phenomenal parts of an overall experience are causally interdependent.

According to WPH, an overall experience consists of phenomenal, sense-modality-specific, parts that are not independent of each other. As a result, the nature of the sensory-phenomenal parts of a total experience depends on the whole. Since the empirical findings of cross-modal interference that support WPH concern our *sense* *modalities,* WPH restricts the dependence to the sensory realm. WPH leaves it open whether a total experience also has proprietary *cognitive* phenomenal parts and, if so, whether they interfere with the sensory phenomenal parts and vice versa. Accordingly, WPH need not apply to all phenomenal parts of a total state, but only to the sensory phenomenal parts, and therefore qualifies as *weak* phenomenal holism.^13^ Since WPH is a weak claim and neither presupposes nor excludes the existence of proprietary cognitive phenomenology, it is a starting point that can be granted by both conservatives and liberals.

One important consequence of WPH is that the sensory phenomenal parts of an overall experience will differ, if subjects differ significantly in the processing of their sense modalities. As a result, the overall experiences of two subjects under the same circumstances can diverge profoundly. (Let me emphasize that WPH does not exclude the possibility that, in many cases, given the same circumstances, the overall experience of an individual A might be a lot like the experience of individual B. It only implies that, *if* two individuals differ significantly in the processing of their sense modalities, their resulting overall experience will differ significantly.)

The following example helps to illustrate the kind of difference that I have in mind. Consider the overall experience of a blind person who is listening to a lively debate. Plausibly, this overall experience differs significantly from the experience of a person under the same circumstances, who can see. Let me clarify that the crucial point here exceeds the trivial claim that the overall experience of listening to a lively debate might differ amongst everybody, due to the different associations and emotions involved.^14^ Rather, the point I want to make is that there will be a *radical* phenomenal difference in the overall experience of individuals who profoundly differ in the processing of their sense organs. As Paul puts it:

Empirically, there is no question that at the most basic conscious level, the first personal experience of the congenitally blind or congenitally deaf is deeply different in kind from first personal experience of sighted and hearing individuals. (2014, p. 69)

Let us pause for a moment to consider where we stand. Empirical research on cross-modal interference points towards weak phenomenal holism which has it that the sensory-phenomenal parts of a total experience are interdependent. This view, abbreviated as WPH, can be granted by both conservatives and liberals and it suffices for the argument developed in the next section. One consequence of WPH is that the sensory-phenomenal parts, as well as the overall experiences, of two subjects will differ radically, if these subjects differ in the processing of their sense organs. In particular, the presence (or absence) of a specific part in one sense modality will radically modify the sensory-phenomenal parts of the overall experience. With this consequence of WPH in mind, let me return to the debate about cognitive phenomenology.

## WPH and Conservatism

If WPH is true, what are the consequences for the competing views about cognitive phenomenology? I start by investigating WPH’s impact on conservatism. If we consider WPH and its consequence of significant interpersonal differences in the sensory phenomenology of conscious thoughts, the limited explanatory power of conservatism will become salient. Let me explain.

First, suppose in accordance with conservatism, that all phenomenology that exists is of the *sensory kind.* Hence, the phenomenology of a particular conscious thought has to be analyzed in terms of sensory phenomenology. Accordingly, in accounting for the phenomenology of conscious thought conservatives rely on the phenomenology of sensations and their analogs in imagery such as inner visual imageries, phonological imagery of inner speech, orthographic imagery of sentences and sensory motor responses. For example, Prinz holds:

We should explain the phenomenology of thought by appealing to inner speech, simulations of what thoughts represent and emotions. The resources can all be characterized as forms of sensory imagery. (2011, p. 192)

Next, consider WPH. According to WPH, an overall experience cannot be analyzed as a mere compound of independent, sense-modality specific, phenomenal parts. Rather, due to the interaction between the sense modalities, the sensory phenomenal parts of an overall experience are intertwined with each other. As a result, the overall phenomenology of a conscious thought, consisting of inner imageries, inner speech, emotions etc. will differ significantly between those individuals who diverge in the processing of their sense modalities.

The following example serves to illustrate the impact of WPH. Take the phenomenology of consciously thinking that *thunderstorms are dangerous.* Presumably, a congenitally blind person will have a different sensory phenomenology tied to this conscious thought than a person who can see. The same will hold for a Deaf person. Importantly, according to WPH, the relevant phenomenal difference is not to be analyzed as the mere lacuna of one particular sensory phenomenology (e.g., the inner image of a lightning strike or of the sound of rumbling thunder). Rather the phenomenal difference is pervasive, by influencing the other sensory phenomenologies as well. The variation in the sensory processing might have an impact on the phenomenology of inner speech, on emotions, etc.—which together result in significantly different overall experiences. Hence, WPH has stronger consequences than the obvious claim that a blind person does not have an inner image of a lightning strike when thinking about thunderstorms—it rather points towards a *profoundly diverging overall phenomenology*. In a nutshell: given WPH, the sensory phenomenology of conscious thought can diverge profoundly among individuals. Does this insight have any significant impact on conservatism? To analyze the impact of WPH, we have to recall that conservatism comes in two flavors.

On *weak* conservatism, the phenomenology of conscious thought is just a *concomitant* phenomenon. Weak conservatism, which has it that there is just a loose connection between sensory phenomenology and conscious thoughts, perfectly fits with WPH. Individuals might differ significantly in their overall sensory phenomenology, but, after all, sensory phenomenology just accompanies conscious thought.

In contrast, according to *strong* conservatism, sensory phenomenology is *constitutive* of conscious thought. That means, it is due to the sensory phenomenology that a particular conscious thought is the thought it is. Now thoughts are commonly held to be individuated by their contents. Therefore, the most plausible way of fleshing out the constitution claim is to hold that phenomenology is constitutive of the (narrow) *content* of a conscious thought.^15^ Since it is generally accepted that two subjects can entertain the same thought content, we have to get clear about the relation between sensory phenomenology and content across subjects. At this point, it becomes clear that WPH poses a challenge to strong conservatism.

## The Challenge for Strong Conservatism

Let me start by considering a version of strong conservatism that *identifies one particular sensory phenomenology* as essential for having a particular target thought. For instance, there is a unique sensory phenomenology φ such that iff one has φ one consciously entertains the thought that *thunderstorms are dangerous.* Such a version of conservatism does not fit with WPH. The reason is that the requirement of an exact one-to-one correlation between a particular sensory phenomenology and a target thought entails that an individual must be capable of having this particular phenomenology to entertain the target thought. Since according to WPH two individuals who differ in the processing of their sense modalities also differ profoundly in their sensory phenomenology, one of them will not be able to entertain the target thought. This is a highly implausible result.

Conservatives might reply that it is very likely that there are some differences in sensory phenomenology between individuals; differences that are the result of different imageries, emotions etc. Therefore, the claim that phenomenology is essential for having a particular conscious thought is to be understood rather as a claim about the relation between sensory phenomenology and conscious thoughts *within one subject*. As Bayne puts this view: “For any subject, S_1_, S_1_’s thought of a certain type (t_1_) has a phenomenal character that sets it apart from S_1_’s thoughts of a different type (t_2_).” (2012, p. 13) On this view, the phenomenology of a particular conscious thought might vary across subjects. Hence, there is no need that everyone has the same sensory phenomenology to be able to entertain a particular thought. It suffices that *for every subject*, there is a particular sensory phenomenology that is uniquely tied to a particular conscious thought. On the assumption that two subjects can entertain the same thought content, this version of strong conservatism fleshes out the relation between sensory phenomenology and the target thought as *multiple realizability.*^16^ That means, the narrow content of a particular conscious thought can be realized by a variety of sensory phenomenologies across subjects. At this point the question arises: to what extent can the sensory phenomenology differ and still be constitutive of the same target thought? As WPH shows, the sensory realizers might be extremely heterogeneous. The main worry is this: how can radically different sensory phenomenologies account in an explanatorily interesting way for having the *same* conscious thought?

Let me elucidate the problem by comparing this version of conservatism with views about *perceptual* experiences with a similar structure. One such view that immediately comes to the mind is functionalism about perceptual experiences. One of the key-claims of functionalism is that perceptual experiences are functional states which are multiply realizable. As the inverted spectra arguments (e.g., Shoemaker, 1982) show, one consequence of functionalism is that the phenomenal character of the experience that realizes the functional state does not matter at all. What accounts for having a particular perceptual experience, e.g., a blue experience, is its functional role (and not it’s blue phenomenal character).

If we compare functionalism about perceptual states to conservatism about conscious thoughts and consider the multiple realizability of a conscious thought by profoundly diverging sensory phenomenologies, the resulting picture parallels functionalism: For having a particular conscious thought, no *particular* sensory phenomenology seems crucial. On this view, it is still essential for having a conscious thought that it is realized in one sensory phenomenology or the other, but these sensory phenomenologies can differ quite significantly.

If so, one might wonder whether it matters at all which sensory phenomenology is involved or whether *any* sensory phenomenology could be constitutive of a target thought. Against the latter view, one could point out that radical divergence does not imply arbitrariness of the sensory phenomenology involved. However, with regard to the specific view discussed here, there are further reasons to think that this view allows that any sensory phenomenology can constitute a target thought.

To see this, let us suppose that the sensory phenomenology involved in thinking a target thought is *not* arbitrary. If so, one might wonder how the restrictions on the sensory phenomenologies that can constitute a target thought can be fleshed out. In particular, one might ask in virtue of which factors these restrictions hold and whether the relevant factor is *intrinsic* or *extrinsic* to the sensory phenomenology.

Notably, in light of WPH, to elaborate on these restrictions via an *intrinsic* factor turns out to be a complicated task. Recall that WPH tells us that the sensory phenomenal parts of an overall phenomenal state are interdependent. Thus, if the sensory processing differs starkly between two subjects, the sensory phenomenologies of subject S1 and subject S2 thinking the same thought can diverge to an extent that they do not share *any* sensory phenomenal feature. Accordingly, in light of WPH, if we consider only factors *intrinsic* to sensory phenomenology, it is hard to flesh out restrictions on the sensory phenomenologies.

This has important implications for strong conservatism. Take the example of having the conscious thought *that thunderstorms are dangerous.* If the sensory phenomenology *alone* constitutes this thought, then the elements in the disjunction of realizers should at least *share some sensory phenomenal* features that account for the fact that these divergent sensory phenomenologies are all realizers of the very same thought. Given the lack of a common sensory phenomenal element, it is hard to see how an aspect intrinsic to these sensory phenomenologies can explain why these sensory phenomenologies are constitutive of the same target thought. The first conclusion of the analysis then is this: Given WPH, sensory phenomenology *alone* is not completely constitutive of conscious thought.^17^ Hence, strong conservatism, which has it that sensory phenomenology alone can explain the constitution of conscious thought, fails.

Next, let me consider the view that some sensory phenomenologies are excluded from the set of possible realizers of a target thought due to an aspect *extrinsic* to the sensory phenomenology. Such a view qualifies as strong conservatism + , since an additional factor is needed to account for the constitution of a target thought. One option would be to hold that some sensory phenomenologies are excluded because they are tied to a particular functional role that makes them inapt for constituting the target thought, assigning an important task to the functional role, rather than to the sensory phenomenology itself. But if the functional facts tie the radically divergent sensory phenomenologies together, in principle any sensory phenomenology could be used, as long as it is tied to the right functional role. For example, a subject S1 might have an inner image of a pink square even when thinking about squirrels, due to some personal, symbolic relation between this sensory phenomenology and the squirrel-content. This seems possible, as long as this inner image fulfills the right functional role. (In Sect. 4.2, I return to this option and analyze one such view in detail.) Therefore, I do not see an objection against the view that, on strong conservatism + , *any* sensory phenomenology can be involved in thinking a target thought.

Now we see the problem which is rooted in the arbitrariness of the relevant sensory phenomenology. The resulting view just has it that sensory phenomenology is essential for having conscious thoughts *in general.* But this does not leave any *explanatory* role for phenomenology when it comes to the constitution of a particular thought content. Rather another, non-phenomenal factor such as functional facts explain why a particular thought is the thought it is. This view, a version of strong conservatism + , fits with WPH since it allows highly divergent sensory phenomenologies. Strong conservatism + assigns a role to sensory phenomenology in the constitution of conscious thought, but one that does not explain why a target thought is the thought it is. The result is that in the light of WPH strong conservatism + fails in its aim to explain the constitution of conscious thought via phenomenology.

To explain the phenomenal constitution of a conscious target thought a common *phenomenal* element has to be found. Conservatives might accept this requirement and flesh out their view in more detail by elaborating on the required common phenomenal feature. Notably, on conservatism, this common element has to be sensory. One option would be the following: First, conservatives might grant divergent sensory phenomenologies by pointing out that the disjunction of the overall phenomenologies that are constitutive of a particular thought might vary along many lines, for instance due to variations in associations, inner imageries or emotions. Second, they might add that there is still one common sensory phenomenal element in all these cases, namely the phenomenology of inner speech of the sentence “thunderstorms are dangerous” that accounts for why these diverging overall phenomenologies are constitutive of the particular target thought. Let me analyze whether this version of conservatism succeeds as a strong CP-thesis.

### Inner Speech

Conservatives might argue as follows: thoughts are commonly held to be individuated by the sentences that are said to “express” them. Accordingly, the sameness of the thought *that thunderstorms are dangerous* can be accounted for by the sameness of the proposition that is articulated in inner speech (or, in the case of a Deaf person, in inner imagery of written words or ASL movements). As long as the phenomenology of the verbal material expresses the same proposition, two persons will think the same thought, so conservatives might say.^18^ Notably, explaining a conscious target thought via the sensory phenomenology of inner speech faces some challenges. The main challenge is to construe inner speech in a way that it both qualifies as purely sensory phenomenology *and* that it explains the phenomenal constitution of thought content.

As Montague (2016) argues, conservatives have to construe inner speech as *low-level* sensory phenomenology, for a more conceptually loaded construal would already imply liberalism. Most conservatives will accept this requirement, but they might add that there are various ways of fleshing out the low-level sensory phenomenology of inner speech.

One option would be to construe inner speech as a mere stream of sound (or line of shapes). One problem with this version of conservatism is that it seems ad hoc to claim that such low-level sensory phenomenology is the only sensory-phenomenal factor that is simply not affected by WPH. More plausibly, the sensory phenomenology of, e.g., an auditory stream will be affected by WPH as well and, hence, can be deeply heterogenous. Conservatives might grant this heterogeneity and hold that the target thought can be realized by a conjunction of diverging low-level inner speech phenomenologies as long as they express the same proposition. But then the question is: due to which factor do these diverging sensory phenomenologies express the same proposition? Since on this view sensory phenomenology is construed as a mere auditory stream, again the decisive factor near at hand is a functional one. If so, the resulting view amounts to an instance of strong conservatism + discussed above. Hence, sensory phenomenology would not *explain* why a particular target thought is the thought it is. The reason is that not phenomenology, but rather a non-phenomenal factor—the functional facts—is key for constituting the thought content.

A second option for conservatives would be to point out that low-level auditory phenomenology need not be construed as a continuous auditory stream but rather is segmented in a particular way. Plausibly, such segmented auditory stream will be affected by WPH as well. Accordingly, the problem with this view is that a segmented auditory stream alone does not suffice to pin down a particular content.^19^ To reach this aim, even a *segmented* sound (or strings of shapes) has to be combined with a further factor that is common to all instances of the same conscious thought. Again, if this factor is fleshed out as a functional one, the constitution of the thought content might be explained, but not by the phenomenology. Hence, the requirement of a strong CP-thesis is not met.

To qualify as a strong CP-thesis, the factor that explains how the diverging low-level phenomenologies of inner speech can constitute the same thought content has to be phenomenal.

One way that conservatives might incorporate a further phenomenal factor would be by understanding the sensory phenomenology of inner speech to be conceptualized, a kind of “verbal material” that already carries a particular meaning. However, such a fine-grained, highly specific conceptualization of auditory phenomenology would amount to cognitive phenomenology and, thus, qualifying, as liberalism. Alternatively, conservatives might hold that the common phenomenal factor is *recognizing or taking* a particular segmented sound as a token of a particular word. While this move might seem promising, it ultimately leads to a dilemma for conservatives. Let me explain. The idea of conceptual taking can be developed in two ways: first, taking-as can be seen as a *phenomenal* factor (Strawson, 2004). If so, this phenomenal way of taking a sentence *as expressing a particular content* requires cognitive phenomenology and qualifies as liberalism.^20^ Second, taking-as can be seen as a non-phenomenal factor. If so, sensory phenomenology does not pin down content, but rather a non-phenomenal factor does. On this view, again, sensory phenomenology is not explanatorily powerful in the sense at issue here.

To sum up the result of the analysis so far: Conservatives might hold that inner speech is the common phenomenal factor that accounts for the phenomenal constitution of conscious thought. Inner speech can be construed as low-level sensory phenomenology, e.g., as a continuous auditory stream or as a segmented auditory stream. In both cases, this low-level sensory phenomenology is plausibly affected by WPH. Moreover, this kind of phenomenology does not suffice to pin down a particular content. Alternatively, inner speech can be seen as already conceptualized in such a fine-grained way to pin down a particular content. However, this view amounts to strong liberalism. Finally, one might hold that inner speech is *taken* as expressing a particular content. This interpretation of inner speech can be developed to support either strong liberalism or a strong conservatism + that assigns the content determining role to functional facts rather than to the phenomenology.^21^ None of these options meets the requirement of strong conservatism, namely to explain in terms of sensory phenomenology why a target thought is the thought it is.

Let me close this Section by pointing out that a conservatism which relies on inner speech faces an additional difficulty. Not all inner speech (or inner orthographic imagery) consists in full sentences. As Siewert argues, there can be experiences of condensed thoughts that do not involve the phenomenology of full sentences:

Suppose you are sitting and reading one morning, and suddenly you remember some incipient appointment—you wonder when exactly it was, feel anxious that you may have missed it, and look at your watch. The thought of the appointment and when it was is an occurrence of consciousness, but it may not be verbalized silently or aloud. You may not have said to yourself—“I have an appointment around now, don’t I? When was it? Did I miss it?” You may not even have said something fragmentary, like: “Appointment! When? Miss it?” […] But this little wordless episode of noniconic thinking—your suddenly recalling that you had an appointment—is phenomenally conscious. (1998, p. 276f)

Moreover, more complex conscious thoughts (e.g., thinking through a philosophical argument or thinking about “philosophical preoccupations and parenthood, and an analogy between their effects” (Siewert, 1998, p. 277)) are unlikely to be fully verbalized as a lengthy, syntactically complex, inner utterance. Thus, the possibility of thoughts without inner speech or the possibility of inner speech that consists only of some key-words popping up in one’s mind, rather than full sentences, additionally challenges the view that inner speech accounts for determinate thought content.

I conclude that pointing at inner speech does not help conservatism to answer the challenge of WPH. Low-level sensory phenomenology of an auditory stream is plausibly affected by WPH and cannot pin down a particular content. High-level sensory phenomenology of either conceptualizing or taking a particular sound as a particular word requires cognitive phenomenology and amounts to liberalism. As Montague rightly points out, to avoid the charge of arbitrariness of sensory phenomenology one has to search for a unifying feature, “but this ‘unifying feature’ is precisely what seems to be missing from the simply sensory approach.” (2016, 196) As we have seen, no such *phenomenal* common factor is available for conservatives.

The remaining option for conservatives is to incorporate a *non-phenomenal* common factor to account for the constitution of conscious thought. Next, I analyze whether such version of strong conservatism + can be fleshed out in a way such that it assigns an explanatorily powerful role not only to the additional non-phenomenal factor, but also to the sensory phenomenology.

### Sensory Phenomenology and Functional Facts

One version of conservatism that incorporates such a unifying, non-phenomenal feature is developed in Pautz (2013). He defends a “phenomenal functionalism” that incorporates sensory phenomenology and functional facts to explain how the content of conscious thoughts can be determinate. According to Pautz, “our actual and potential sensory experiences, together with the functional facts, are enough to secure our actual level of content determinacy.” (2013, p. 201).

At first glance, this seems to be a promising approach to explain content determinacy via sensory phenomenology. However, if we consider phenomenal functionalism carefully, it turns out that there are various ways of interpreting this view and none of them meets *both* requirements of a strong version of conservatism. On one reading, the requirement that phenomenology is key for content determinacy is met, but phenomenology turns out to be proprietary cognitive phenomenology. On another reading, the requirement to invoke only sensory phenomenology is met, but it turns out that functional facts are key for content determinacy, rather than phenomenology. To see this, let us look closely at the way how Pautz describes the sensory phenomenology and functional facts that, on his view, constitute the belief *That is a round tomato*:

(S)uppose (a man) currently has an experience of a round tomato, the sentence “that’s a round tomato” runs through his inner speech and he also assents to this sentence in public speech; and he manifests understanding this sentence. […] No one competent with the concepts of belief and desire would deny that […] he believes a round tomato is present. (2013, p. 203).

Considering this description, how should we understand the sensory phenomenology involved?

Let me start with the sensory phenomenology of having “an experience of a round tomato”. This can be read in two ways: on one interpretation, the sensory phenomenology is high-level phenomenology. There is controversy about whether high-level properties can be represented in perception (additionally to being represented in judgements).^22^ If one thinks that high-level perception is possible, the resulting view involves conceptualized sensory phenomenology. After all, without the relevant concept, one cannot see a tomato *as a tomato*. Plausibly, having an *inner image* of a round tomato, then, is high-level sensory phenomenology that is constituted by a tomato-concept. Most liberals hold that such high-level sensory phenomenology qualifies as liberalism. For example, Montague argues in the following way that granting such high-level phenomenology amounts to liberalism: “(1) The representation of high-level properties requires the deployment of concepts. (2) The deployment of concepts is necessarily associated with cognitive phenomenology. (3) Therefore, the representation of high-level properties is necessarily associated with cognitive phenomenology (1, 2). (4) When high-level properties are represented in perception, they are necessarily associated with cognitive phenomenology (2, 3).” (2018, pp. 10–11).

Conservatives who deny that the deployment of concepts necessarily involves cognitive phenomenology might opt for another interpretation of Pautz’s quote to illustrate their view. For example, having “an experience of a tomato” can be read as having a low-level, non-conceptual sensory phenomenology and a deployment of the “tomato” concept in a judgement. On this interpretation, having an experience of a tomato fits with conservatism. However, as a consequence, the low-level sensory phenomenology does not explain the determinate content but rather the concept application in the judgement does.

Next, besides “having an experience of a round tomato”, Pautz also mentions having the experience of the sentence “that’s a round tomato” running through the man’s inner speech. What can we say about this? The results of the analysis in Sect. 4.1. apply also to this instance of inner speech: Referring to conceptualized *inner speech* that already carries a specific meaning clearly involves cognitive phenomenology. Such phenomenology is promising to secure content determinacy and could also explain why it is tied to certain functional facts. However, the view departs from the key tenet of conservatism that only sensory phenomenology exists. Alternatively, one might conceive of the inner speech as a low-level, parsed auditory stream. Such low-level sensory phenomenology cannot secure determinate content. Accordingly, what allegedly determines the content then has to be the functional facts, e.g., that the man “assents to this sentence in public speech and manifests understanding this sentence”. Given that this second interpretation assigns priority to functional facts, it is compatible with the insight of WPH that sensory phenomenology can be deeply heterogenous. Low-level sensory phenomenology then still might contribute to the constitution of content, but only by serving as a (often highly divergent) basis to which the decisive function is tied. Importantly, no *particular* sensory phenomenology is needed for having the target thought. The resulting view qualifies as a strong conservatism + that incorporates sensory phenomenology as one element of conscious thought, but one that does not explain content determinacy. Rather functional facts explain why a target thought is the thought it is.

Along with Pautz’s description of having a conscious belief, the key claims of his view concerning phenomenal functionalism only make sense on these two interpretations (viz., that it either involves cognitive phenomenology or assigns the explanatory role to the functional facts). For example, Pautz holds that “some sensory-functional conditions are a priori incompatible with having that belief or desire. In this sense, there are *sensory-functional constraints on belief and desire*.” (2013, p. 213).

The notion of “sensory-functional” allows multiple readings. With regard to the sensory phenomenal part, these constraints might be true if sensory phenomenology is taken to be specifically conceptualized, i.e., *cognitive* phenomenology. Again, this view qualifies as liberalism. In contrast, if the sensory phenomenology is taken to be low-level, I do not see the necessity of such constraints. Functionalism traditionally allows for multiple realizations and, in extreme cases, even seemingly inapt realizers can still fulfill this function. This fits well with the lesson from WPH that two persons can have the same conscious thought although their sensory phenomenologies radically differ. Accordingly, on the second interpretation, the restrictions are due to the functional facts. Sensory phenomenology, again, is no longer key.

Now one might object that I have not considered the *combination* yet, namely that the constraints consist in the functional facts which can be identified only by their relations to low-level sensory phenomenology. Pautz specifies his view as follows: “Karl’s experiences of the world, which determine his history of evidence, are anchor points that help to determine, via the humanity constraint, the contents of his downstream beliefs […], by ruling out deviant interpretations” (2013, p. 225). Considering this specification of the combination, the view again only makes sense if fleshed out as either liberalism or as a strong conservatism + that assigns the explanatory role to the functional facts. First, it is hard to see how low-level sensory experiences (e.g., an inner imagery of a particular shape or sound) can serve as such anchor points. If phenomenology provides anchor points that are needed to determine content, sensory phenomenology has to be taken as high-level and conceptual and, hence, the resulting view qualifies as liberalism. Second, if only low-level sensory phenomenology is part of the combination, it is again functional facts that determine content.^23^ In short: on each interpretation, phenomenal functionalism turns out to be either a strong liberalism or a version of strong conservatism + on which not sensory phenomenology but rather functional facts *explain* content determinacy.

Let me summarize the result so far. We wanted to find out whether conservatism is compatible with WPH. First, it turned out that weak conservatism is compatible with WPH but only because this view reduces phenomenology to a concomitant phenomenon of conscious thought.

Next, we considered strong conservatism and its aim to account for the constitution of conscious thoughts via sensory phenomenology alone. It turned out that WPH poses a serious challenge to strong conservatism. Given that two subjects can entertain the same thought, a particular conscious thought could be constituted by a disjunction of radically diverging sensory phenomenologies. Hence, there must be a common factor which secures a determinate thought content.^24^ Recall that according to conservatism only sensory phenomenology exists, and hence there is no common *phenomenal* factor left that could be unaffected by WPH.

Finally, we considered strong conservatism + , which has it that sensory phenomenology together with another, non-phenomenal, factor constitutes conscious thought. Strong conservatism + fits with WPH. However, in light of WPH, it turned out that it has to be the additional, non-phenomenal, (e.g., a functional), factor that accounts for why the radically diverging sensory phenomenologies are realizers of the same thought. Thus, strong conservatism + leaves no interesting role for sensory phenomenology when it comes to *explaining* determinate conscious thought.

The result of our analysis is that given WPH, conservatism cannot account for the *phenomenal constitution* of conscious thought. Defenders of weak conservatism will agree with that result since they reduce the phenomenology of conscious thought to a concomitant phenomenon at the outset. Strong conservatism aims at explaining the constitution of conscious thoughts via sensory phenomenology. Unfortunately, given WPH, strong conservatism and strong conservatism + both fail in this aim. Hence, if one thinks that phenomenology is explanatorily powerful when it comes to the constitution of conscious thought, one has to abandon conservatism about cognitive phenomenology.

## WPH and Liberalism

Next, let me investigate whether liberalism fits better with WPH. Recall that liberalism comes in two strengths. On liberalism*, proprietary cognitive phenomenology is *modally independent* of sensory phenomenology.^25^ That means, even an individual with no sensory phenomenology at all could enjoy a phenomenology of conscious thoughts.^26^ In contrast, on liberalism + , proprietary cognitive phenomenology is *irreducible* to sensory phenomenology. Liberalism + allows that the sui generis cognitive phenomenology involves (or depends on) sensory phenomenology.

Both kinds of liberalism typically aim at a strong CP-thesis and, hence, defend the claim that a proprietary cognitive phenomenology is constitutive of conscious thought. At this point, one might wonder whether liberals face a similar challenge due to WPH as conservatives. Recall that WPH points towards a multiple realizability of a particular conscious thought by a variety of sensory phenomenologies. That means two subjects can diverge profoundly in their sensory phenomenologies while entertaining the same conscious thought. The challenge that WPH poses to every strong CP-thesis is: to what extent can the overall phenomenology vary across subjects and still account in an explanatorily interesting way for the constitution of a particular thought? Liberals can deal with this challenge in various ways.

### Liberalism*

Consider first liberalism*. Liberals* can easily incorporate WPH into their view that a proprietary cognitive phenomenology is constitutive of conscious thought. They can point out that WPH is restricted to sensory phenomenology and need not equally affect the proprietary cognitive phenomenology. After all, we only have empirical evidence about phenomenal holism concerning the cross-modal processing of our *sense modalities.* Since on liberalism* proprietary cognitive phenomenology is of a sui generis kind that is modally independent of sensory phenomenology, WPH need not apply to it. Subjects vary in their overall sensory phenomenologies, but they have the very same proprietary cognitive phenomenology when they entertain the same conscious thought, liberals* might say.

This claim can be further spelled out in two ways. First, one might hold that a cognitive experience is a purely cognitive-phenomenal state without any sensory-phenomenal aspects. Alternatively, liberals* might allow that a conscious thought often consists of both sensory -phenomenal and cognitive- phenomenal aspects and add that the latter is unaffected by the former. Hence, the cognitive-phenomenal aspect is the stable common phenomenal factor we are searching for. Holism then turns out to be “local holism” about sensory phenomenology, which liberals* can combine with atomism about proprietary cognitive phenomenology. This way of dealing with WPH could be adopted, for example, by liberals like Pitt who tells us:

once it is accepted that our thoughts have a distinctive sort of phenomenology that is content-constitutive, […] the intuition that there may be many different ways to think that p ought to be explained away in terms of the different phenomenologies of other, non-cognitive types (e.g., visual, auditory, emotional, etc.) that might accompany episodes of conscious thinking for other people, or for creatures other than humans. (2009, p. 133f.)

In short: Liberals* can add WPH to their version of a strong CP-thesis, since WPH need not affect the proprietary phenomenology which according to liberalism* is key for the constitution of conscious thoughts.^27^ That means, WPH does not even pose a challenge to liberalism*. However, to some theorists, liberalism* might seem an ad hoc response to the challenge by WPH. Moreover, it rests on the controversial premise that a proprietary cognitive phenomenology exists that is modally independent from sensory phenomenology.^28^

### Liberalism + 

On liberalism + , WPH plausibly affects the *overall* phenomenal state of entertaining a conscious thought because this state is characterized as involving sensory-phenomenal *and* cognitive-phenomenal aspects. Notably, this does not pose a serious challenge to liberalism + .

One option for incorporating WPH into liberalism+  goes as follows. First, liberals+ can grant that the proprietary cognitive phenomenology also depends on the overall experience and, hence, can vary across subjects having a conscious target thought. They might add that despite these variations, the divergent experiences display important similarities among each other that suffice to account for the same narrow content. In contrast to sensory phenomenology, there is no empirical evidence for *significantly* diverging proprietary cognitive-phenomenal components of experiences between subjects. Hence, liberals + are free to hold that—although some aspects of cognitive phenomenology might vary across subjects as result of being a component of diverging overall experiences—there is still a similarity in the cognitive phenomenology that is given in all instances of the same conscious thought. For example, Pitt mentions, but does not endorse, the view that “phenomenal types are to some degree vague, and that token conscious states that differ in their phenomenology might nonetheless be of the same (vague) type.” (2009, p. 134) To many theorists this view will seem unattractive, for it rests on the premise that vague cognitive-phenomenal types exist that are constitutive of conscious thoughts. Therefore, in the final section, I will propose another model of liberalism + that incorporates WPH, rests on less controversial premises, and that is explanatorily powerful with regard to the phenomenal constitution of conscious thoughts.

## A Proposal: *The fusion account*

If one thinks that phenomenology is constitutive of conscious thought in the sense of determining the thought content, in one way or another concepts have to be integrated into this view.^29^ There are several ways to flesh out the role concepts can play with regard to phenomenology. I will briefly discuss three options which all face the challenge posed by WPH. Then I will propose a version of liberalism + that incorporates a conceptual factor as well but fits better with WPH.

- Option (i): *Causal* impact of concepts on sensory phenomenology.

Let us start with the plausible assumption that we possess the capacity to apply semantic properties to sensory phenomenology. The execution of a capacity which uses a phenomenal state as its basis will likely result in a further phenomenal state. However, philosophers disagree about whether such concept deployment leads to a *new* kind of phenomenology, over and above sensory phenomenology. For example, Carruthers and Veillet (2017) think that concepts merely have a *causal* impact on sensory phenomenology, but phenomenology-wise the result of this impact is again sensory phenomenology. Defending a causal impact of concepts on sensory phenomenology leads to conservatism, which in its strong version faces the challenge analyzed above.

- Option (ii): *Constitutive* impact of concepts on sensory phenomenology.

On another view, concepts have a *constitutive* impact on sensory phenomenology and the result of this impact is *high-level* sensory phenomenology. Since according to some theorists (e.g., Bayne, 2009; Siegel, 2010; Siewert, 1998), perceptual experiences can have high-level content, such high-level experiences could be a necessary part of conscious thought too.

Many liberals think that if concepts partly *constitute* sensory phenomenology, it necessarily involves cognitive phenomenology and liberalism+ is established (see, e.g., Montague, 2016, p. 178; 2018, pp. 10–11). I agree that such a view qualifies as liberalism+. However, depending on the view one endorses with regard to sensory phenomenology in general, some conservatives might question this qualification. In any case, the main worry is that WPH poses a challenge to the high-level sensory phenomenology view as well. The challenge is: how profoundly can the low-level sensory-phenomenal basis diverge and still be the basis for the same high-level content? Plausibly, there will be restrictions to the kind of low-level sensory phenomenology that can serve as basis. For instance, the low-level visual phenomenology of a pink square is a bad candidate for giving rise to a high-level, sensory, “squirrel”-experience. Thus, the high-level sensory phenomenology view faces again the challenge by WPH, but it shifts this challenge one level down. Now the problem is not to explain the relation between high-level sensory phenomenology and content, but rather to explain how the radically divergent low-level sensory phenomenologies can serve as basis for the same high-level sensory phenomenology so that it accounts for the same thought content.

- Option (iii): Concepts constituting a *thematic unity.*

On a further account, developed by Nes (2011), the phenomenology of conscious thoughts exhibits a thematic unity, and conceptual content is the factor that constitutes this thematic unity.^30^ On this account concepts are crucial, but they do not cause or constitute sensory phenomenology. They rather constitute the thematic unity around which the various sense-modality specific phenomenologies revolve. I think Nes’s account gets a lot right about cognitive phenomenology. However, it also faces the challenge of explaining how radically sensory phenomenologies can diverge and still be unified around the same theme.

Since all these accounts face the challenge of WPH, let me suggest a further view about the role concepts play in cognitive phenomenology, which fits better with WPH. Here is my proposal: applying concepts to sensory phenomenology generates a *new, hybrid*, *phenomenal state*. Let me explain.

First, sensory phenomenology is the basis for the hybrid phenomenal state. Second, the sensory phenomenology is taken in a specific way and it is thereby fused with concepts. The result of the fusion is a new phenomenal state with a hybrid character that has two aspects: a sensory-phenomenal one and a cognitive phenomenal one. These two aspects are not just co-occurring, independent, features but rather fused and thereby bring about the new phenomenal state. The key aspect of this hybrid state is the cognitive phenomenal one. The sensory-phenomenal aspect is a necessary aspect of this hybrid state, but there are no restrictions on the specific *kind* of sensory phenomenology. Thus, the result of the fusion is not a high-level sensory phenomenology as analyzed above, since high-level sensory phenomenology is commonly conceived as placing heavy restrictions on the kind of low-level phenomenology that can serve as its basis.

The phenomenal character of the hybrid state can be captured by the notion of the *what-it-is-likeness to take sensory phenomenology to represent a specific content*. This way of putting is borrowed from Strawson. He characterizes conceptual intentionality as

a feature of any experience that involves apprehending something as being of a certain kind, as an F, say, or as G or H; any experience, in other words, that involves deployment of a concept, bringing a thing ‘under’ a concept […]. If there is ‘as-ness’, if there is any kind of taking-of-something as-something that is experientially realized in any way (e.g., in any conscious thought that involves deployment of a concept), if the ‘as’ phrase is truly appropriate in the description of a mental phenomenon, then we have conceptual intentionality. (2004, p. 305)

I think Strawson is right in pointing at the experiential way of deploying concepts to account for cognitive phenomenology. Thus, the fusion account draws upon his taking account. But the fusion account also diverges in the following respects from Strawson’s.

First, I propose a broader application of Strawson’s claim that an *object* can be taken as an F. I suggest that not only objects, but also an overall *sensory phenomenology* can be taken to stand for a specific content. The taking-as fuses the overall sensory phenomenology with concepts in a way that generates a new, hybrid, phenomenal state.^31^

The reason for focusing on the overall sensory phenomenology is the following: the fusion account aims at explaining the phenomenal constitution of conscious thought via its narrow contents. If we consider whole propositions such as “*thunderstorms are dangerous”*, the phenomenal constitution of this content can be fleshed out in two ways. First, one might think that the sensory phenomenologies tied to each word (expressing the concept) is taken in a specific way and the succession of these single takings gives rise to entertaining the relevant content. Second, one might hold that there is an *overall sensory phenomenology*, for example, an inner image of a scene of a thunderstorm (and perhaps the emotion of feeling frightened and a motor response of tension etc.) that is taken to stand for the whole content that *thunderstorms are dangerous*. The latter interpretation is more plausible. To see this, consider the case from understanding ambiguous sentences (Horgan & Tienson, 2002; Pitt, 2004; Siewert, 1998). The mere succession of sensory phenomenologies each tied to a single word does not account for the different understanding experiences. Thus, if one holds that the phenomenology involved in conscious thought is inner speech, the mere succession of the inner speech elements cannot explain the two different thought contents. Rather, there has to be an overall phenomenology of taking the complex sensory phenomenology in a particular way. This is exactly what the hybrid phenomenal state amounts to.

Moreover, one might hold that the phenomenology of thought is not inner speech but rather inner visual imagery. Notably, there is nothing in inner imagery that restricts it to stand only for single objects. Inner visual imagery is rich, and besides being taken to stand for a single object, it can also be taken to stand for an event or a state of affairs. If the sensory phenomenology tied to a particular thought content is inner visual imagery, taking the overall sensory phenomenology in a specific way seems the more plausible account than referring to a succession of three takings of three inner images (of thunderstorms, of “are” and of danger). In short: what speaks in favor of taking an overall sensory phenomenology in a particular way is that it fits well with the richness of visual imagery and that in the case of inner speech it also explains ambiguous sentences. It is an advantage of the fusion account that its crucial element—the hybrid phenomenal state—can also explain these cases that are hard to make sense of on taking-accounts that rely only on taking single items.

The second feature that differentiates my proposal from Strawson’s account is that the connection between the sensory phenomenology and the conceptual taking is tighter on the fusion account.^32^ These aspects are intertwined with each other and unified to a new, hybrid, experience of what it is like to think a particular thought.^33^

The fusion account qualifies as a strong CP-thesis, since on this view a conscious thought has its contents in virtue of its phenomenology. The phenomenology in question is a hybrid one that has two aspects: a sensory phenomenology and a conceptual taking of this sensory phenomenology in a particular way. Since sensory phenomenology is a necessary aspect of the hybrid state, it qualifies as liberalism + . How does this view deal with the challenge posited by WPH?

Given WPH, two subjects can have radically divergent sensory phenomenologies but entertain the same thought. Hence, if there is a phenomenal sameness in having a particular conscious thought, it cannot be the sameness of sensory phenomenology. Thus, there must be another common phenomenal factor figuring in all conscious tokenings of the target thought. I suggest that the common factor is found in the phenomenal taking-as and, hence, in the fusion of concepts with the sensory phenomenology. Accordingly, radically diverging sensory phenomenologies can be the basis for a particular conscious thought insofar as they figure as an aspect of a hybrid state that intertwines them with the relevant semantic properties. Therefore, the hybrid phenomenal states of two subjects having the same thought might differ in the sensory phenomenal aspect, but they also share a decisive common phenomenal factor. Let me emphasize that I do not mean that the common factor is the applied *concepts themselves*. The common factor, which is not affected by WPH, is a phenomenal one: it is the phenomenal taking-as of the diverging sensory phenomenal manifolds.

The fusion account allows a high variation of the sensory phenomenology. On the one hand, profoundly *diverging* sensory phenomenologies can partly constitute the *same* conscious thought. On the other hand, the *same* sensory phenomenology can constitute *different* conscious thoughts. Let me say more about each case.

First, one might wonder why the fusion account allows this extreme variation of the sensory phenomenal basis. After all, in the discussion of the high-level account, the necessity of constraints on the relevant low-level sensory phenomenology was emphasized. This consideration helps to highlight the differences between the fusion account and this rival account. As Siegel’s (2010) famous example of a visual experience of a pine tree shows, high-level sensory experiences are the result of an expertise to recognize low-level properties that are crucial for the relevant high-level content. Accordingly, the low-level sensory basis can vary in some respects, but still has to contain at least some of these crucial features. In contrast, on the fusion account, the taking-as is not necessarily restricted to a particular set of sensory phenomenologies. Although in most cases, the sensory phenomenal basis will share some crucial features, in principle it is possible that also a seemingly inapt low-level sensory basis can be taken to stand for a particular content. The following example, which is based on different processing of the sense modalities, helps to illustrate this point. Consider a congenitally blind person who has the conscious thought *The office is at the end of the hall.* As studies show (e.g., Thaler et al. 2011), besides tactile phenomenology, blind individuals strongly rely on auditory phenomenology to navigate in space. Accordingly, some blind individuals develop excellent echolocation skills to orient themselves in the environment. Moreover, recent studies (Gori et al., 2020a, b) show that congenitally blind individuals use temporal representation of events to represent spatial metrics. Using a temporal coordinate system, they develop auditory maps for spaces, such as home or office buildings. Considering this, the overall sensory phenomenology of a blind person thinking *The office is at the end of the hall* might radically differ from that of a sighted person having the same thought.^34^ On the proposed account the phenomenal taking-as is the decisive factor and, hence, in principle, any sensory phenomenology can be taken to stand for a particular content. Now it becomes clear that this is an advantage of the view: it accounts for the sameness of thoughts across individuals also in cases in which the sense modalities are processed differently and the resulting sensory phenomenologies of two persons diverge radically.^35^

Second, one might wonder how the *same* sensory phenomenology can constitute *different* conscious thoughts. Recall that the sensory phenomenology is very rich and, hence, can serve as a basis for many thought contents. Take, for instance, the sensory phenomenology of an inner image of a thunderstorm. It can be used to represent *that there is a thunderstorm approaching*, *that this is a frightening weather condition, that this brings the much-desired rain after a drought* etc. The particular content that this sensory phenomenology partly constitutes is pinned down by the concepts that are fused with it via phenomenal taking.^36^ Hence, in both cases, the dependence of cognitive phenomenology on sensory phenomenology is a rather weak one, for it suffices that there is *some* kind of sensory phenomenology. In principle, there are no constraints on the particular kind of sensory phenomenology.^37^

At this point, one might wonder why some sensory phenomenology is needed at all for having a particular conscious thought? Since the fusion account allows arbitrariness of sensory phenomenology, the particular sensory phenomenology involved does not contribute in an explanatorily interesting way to the constitution of the target thought. Accordingly, if one thinks that liberalism is true, why not choose liberalism* and hold that cognitive phenomenology is completely independent of sensory phenomenology?^38^

I agree that sensory phenomenology does not explain the phenomenal constitution of a target thought. This is the result of the analysis of strong conservatism and strong conservatism + , and it equally applies to the fusion account. However, in contrast to strong conservatism + , the fusion account offers another *phenomenal* element (rather than the non-phenomenal, functional one) that explains the constitution of conscious thought—namely, the phenomenology of taking the sensory phenomenology in a particular way.

The role of sensory phenomenology then is the following: although sensory phenomenology does not *explain* the constitution of conscious thought, it is still one necessary aspect of conscious thought. Some sensory phenomenology is needed, since the conceptual taking has to take *something* to stand for a particular content. In other words: the sensory phenomenology is a necessary substratum without which the phenomenal taking cannot get off the ground. The proposed view accounts for the intuition that some sensory phenomenology is always present in conscious thought and it is compatible with the insight of WPH that the sensory phenomenology can be deeply heterogenous. This means that the fusion account denies the key claim of liberalism* that we can have a conscious target thought in the absence of sensory phenomenology, but it agrees with liberalism* that sensory phenomenology does not contribute in an explanatorily interesting way to the constitution of conscious thought. Thus, those who share the intuition that conscious thought always involves some sensory phenomenology will prefer the fusion account over liberalism*. However, if one thinks that pure cognitive phenomenology is not controversial, one might choose liberalism*. Both views explain the phenomenal constitution of conscious thought and both views deal with the challenge of WPH in a satisfactory way.

Let me close by highlighting the explanatory power of the proposed view. First, the fusion account clearly qualifies as a *strong* CP-thesis, for it explains the constitution of conscious thought via (cognitive) phenomenology. Importantly, as a strong CP-thesis it has a solution to the challenge due to WPH, viz. it answers the puzzle of how profoundly diverging sensory phenomenologies can (partly) constitute the same thought. Second, it explains the reverse cases as well, viz. how the same sensory phenomenology can constitute different thoughts. Given the richness of sensory phenomenology and the connected content-indeterminacy problem, two subjects can have the same sensory phenomenology and different thoughts.^39^ Thus, conscious thought does not supervene on sensory phenomenology. The overall phenomenology of entertaining different thoughts varies, for it results from a fusion of the (same) sensory phenomenology with diverging cognitive-phenomenal takings. In this way, the fusion account provides us also with a necessary condition for having a particular thought. If a subject S *phenomenally takes* a particular sensory phenomenology to stand for content p, then S necessarily consciously thinks that p.

In short: the fusion account is attractive for at least three reasons. First, it successfully incorporates WPH in a strong CP-thesis—a task that cannot be accomplished by conservatism. Second, it is less controversial than liberalism* since it does not presuppose that cognitive phenomenology is completely independent of sensory phenomenology. It grants that the phenomenology of thought necessarily involves some sensory phenomenology but it emphasizes that the cognitive-phenomenal aspect of taking neither requires nor is influenced by a particular sensory phenomenology. Third, among the extant versions of liberalism + , the fusion account deals better with the challenge of WPH than, for instance, the high-level constitutional view, and it does not rest on the controversial premise that vague phenomenal types exist.

## Conclusion

The cognitive phenomenology debate centers on two questions: (1), what is an apt characterization of the phenomenology of conscious thought? And, (2), what role does this phenomenology play? It turned out that the answers to the former question bear heavily on the possible answers to the latter question. In particular, conservatism is not compatible with the strong CP-thesis that aims at explaining the constitution of conscious thought via phenomenology. To determine this, we had to get clear about sensory phenomenology. I approached this issue from a new angle that has not received much attention in the literature yet. By considering weak phenomenal holism about sensory phenomenology, I focused on profoundly diverging sensory phenomenologies. Next, I elaborated on a challenge that WPH poses for strong CP-theses, namely: can radically diverging sensory phenomenologies explain the constitution of a particular conscious thought? Considering WPH brought clarity to the question which kind of phenomenology can explain the constitution of conscious thought.

It turned out that strong conservatism and strong conservatism + cannot deal with the challenge of WPH in a satisfactory way. Due to the possibility of radically diverging sensory phenomenologies there is no *phenomenal* common factor left that could secure content determinacy. This significantly reduces the explanatory power of conservatism by leaving no interesting role for sensory phenomenology in the constitution of conscious thought.

In contrast, WPH does not pose a serious challenge to liberalism. Since the empirical evidence for WPH only concerns sensory phenomenology, on liberalism* WPH need not affect proprietary cognitive phenomenology. However, liberalism* rests on the controversial premise that cognitive phenomenology is modally independent of sensory phenomenology.

On liberalism + , the overall phenomenology of having a conscious thought is plausibly affected by WPH since it is a combination of sensory-phenomenal and cognitive-phenomenal features. I outlined a novel version of liberalism + , the *fusion account*, that answers the puzzle of how an overall experience, that involves profoundly diverging sensory phenomenologies, can constitute the same conscious thought. The solution is found in a stable cognitive-phenomenal factor of a new hybrid phenomenal state—namely, the phenomenal way one takes the diverging sensory phenomenology to stand for a particular thought content. I conclude that if one thinks that phenomenology explains why a conscious thought is the thought it is, one should endorse liberalism about cognitive phenomenology. In particular, the fusion-account does justice to WPH and offers a promising framework for understanding the phenomenal constitution of conscious thought.

## Footnotes

1 For capturing the what-it-is-likeness of a conscious state (Nagel 1974), I use the notions “phenomenal character” and “phenomenology” and I use these notions interchangeably. The notion “phenomenology of thought” should be read as an abbreviation for the phenomenal character of occurrent conscious cognitive states such as beliefs, intentions, thoughts etc.

2 Within the debate about cognitive phenomenology, the labels “conservatism” and “liberalism” are introduced by Bayne and Montague (2011).

3 Such phenomenal contrast scenarios are put forward in Strawson (1994), Horgan and Tienson (2002), Horgan and Graham (2012), Pitt (2004), Siewert (1998), Chudnoff (2015). For example, Pitt (2004) uses garden-path sentences or center-embedded sentences to induce in the reader a phenomenal contrast between the initial puzzlement and the experience of understanding these sentences.

4 For an explanation of the steadfastness of both sides of the debate in the face of ostensive phenomenal contrast arguments, see Fürst (2017).

5 Another way to argue for liberalism is to describe scenarios in which a subject entertains a phenomenally conscious thought, but there is *no* sensory phenomenology involved. Examples include Kriegel’s (2015) Zoe-argument and Siewert’s (1998) discussion of non-iconic thought. A critical discussion of such cases is found in Pautz (2013).

6 Since the aim of the paper is to analyze competing cognitive *phenomenology* theses, I focus on the constitution of *conscious* thought. For the lack of space, I cannot discuss unconscious thought and its relation to conscious thought. Let me just mention two ways to flesh out the importance of phenomenology for unconscious thought. One might think that we can have phenomenal states we are not aware of (Pitt 2009) and unconscious thought could be such state. On another view, unconscious thought is derivative of conscious thought. For example, Bourget (2010) holds that standing states derive from connections to occurrent thoughts, which are phenomenal intentional states. Kriegel’s (2011) interpretivism, according to which standing or unconscious states derive from an ideal interpreter’s phenomenal states, qualifies as a derivative view as well. My aim here is to analyze conscious thought, thereby providing the first step to a full understanding of thought in general.

7 For a helpful taxonomy of cross-modal experiences, see MacPherson (2011.)

8 For a discussion of studies about cross-modal interferences, see O’Callaghan (2008).

9 I use the notions “phenomenal state” and “experience” interchangeably.

10 The qualification “overall” or “total” experience is used to refer to a complex*, multimodal*, phenomenal state. I leave it open whether *all* local experiences *necessarily* combine into one overall experience (as Bayne and Chalmers (2003) suggest in their “phenomenal unity thesis”) or whether only some of them—for instance, the *sensory* ones—do.

11 Strong dependence can be interpreted in two ways—as the claim that the overall experience is more *fundamental* than its parts or as the *modal* claim that the parts would not exist if they were not parts of the overall experience (Koksvik 2014).

12 For an illuminating analysis of how the relation between experiences and their parts can be fleshed out, see Lee (2014.)

13 Some might think that WPH is weak to the extent that it can be granted even by defenders of weak atomism. For example, Lee thinks that weak atomism is compatible with what he calls “local holism”. “The experience of the shape, color and texture of a surface could be holistically interdependent, even though typically an experience does have *some* independent parts (parts whose existence and nature do not depend on other experiences)” (2014, p. 298) That means if one thinks, as Lee does, that “weak atomism” is the view that at least *some* phenomenal parts are independent of a total phenomenal state, WPH is compatible with weak atomism.

14 Montague makes this point when she tells us: “(I)t’s hugely implausible that you have to have an image of green grass, or a patch of green, in order to consciously think that grass is green. At the very least, there is presumably some possible variation in what these images can be. John could have various associations with grass.” (2016, p. 194) I am concerned with more radical variations of sensory phenomenology which serve to illustrate the seriousness of the challenge due to WPH.

15 Restricting the constitution claim to the attitude type of a conscious cognitive state would not give us the desired result of phenomenology being essential for having a *particular* conscious thought.

16 As an anonymous referee points out, one option would be to restrict strong conservatism to a view that only explains the constitution of *token* conscious thoughts. Conservatives might hold that since WPH is empirically supported, it suggests that there is no type individuating of conscious thought that p across individuals after all. This is an interesting option for conservatives. However, the intuition that two persons can entertain the same thought is widely shared. Accordingly, if—as I will argue—liberalism can grant WPH *and* explain the occurrence of a particular conscious thought across individuals, liberalism will be explanatorily more powerful. This would be a significant advantage of liberalism over the “token” version of conservatism.

17 Thanks to an anonymous referee for drawing my attention to this point.

18 Thanks to an anonymous referee for drawing my attention to this line of thought.

19 A clarification is needed here. A segmented auditory phenomenology can help recognizing certain features of an utterance or of the speaker. Consider an utterance of a newscaster. A hearer who does not understand the language the newscaster is speaking might be able to recognize it as an utterance in German due to the way the auditory stream is segmented. Moreover, a hearer who is familiar with the timbre of the newscaster might be able to recognize her identity due to such low-level, segmented auditory phenomenology. (Thanks to an anonymous referee for drawing my attention to this.) Accordingly, I do not deny that low-level sensory phenomenology of a segmented auditory stream can provide us with important general information such as the language or identity of the speaker. However, the crucial point is that such low-level auditory phenomenology cannot pin down a determinate content.

20 I develop a taking-account that qualifies as liberalism in Sect. 6.

21 An anonymous referee notes that the sensory phenomenology (e.g., the auditory stream) might be needed as a realizer of the functional role. This might well be the case. The point here is that sensory phenomenology then does not *explain* why the target thought is the thought it is. Rather, it is the functional role, which can be realized by radically diverging sensory phenomenologies, that is explanatorily powerful in this respect.

22 For a defense of the view that high-level properties can be represented in perception, see e.g., Bayne (2009), Siegel (2010), and Siewert (1998). For criticism of this view see, e.g., Brogaard (2013), Prinz (2013), and Tye (2000).

23 I agree with Pautz that the phenomenology of thought is not modally independent from all sensory phenomenology. However, the crucial question is: which factor is decisive for constituting thought content and which one serves as a—presumably necessary, but still arbitrary—basis? The main lesson learned from WPH is that sensory phenomenology can be deeply heterogenous. Hence, sensory phenomenology is an obvious candidate for serving as such an arbitrary basis, whereas another factor accounts for why two persons with radically different sensory phenomenologies can have the same conscious thought. Also on the second, combinatory reading of phenomenal functionalism, functional facts are decisive and, hence, content determinacy is secured by a non-phenomenal factor.

24 The problem is not whether a particular thought is *compatible* with a variety of sensory phenomenologies. (It is). Rather, I am concerned with the question whether radically diverging sensory phenomenologies can *explain* determinate conscious thought.

25 Defenders of liberalism* are e.g., Kriegel (2015), Pitt (2004), Chudnoff (2015). Siewert’s (1998) arguments of non-imagistic thoughts could be also used to establish liberalism*.

26 The key-claim of liberalism* is illustrated by Kriegel’s “Zoe-argument” (2015, p. 55ff) which aims to establish a sui generis, proprietary, cognitive phenomenology. Kriegel asks the readers to conceive of “Zoe”, a hypothetical person who is deprived of all kinds of sensory phenomenology. Zoe spends her time running mathematical proofs. According to Kriegel, we can conceive of a phenomenal contrast between Zoe’s mental state of not getting a mathematical proposition and her mental state of grasping it later. Hence, sensory phenomenology seems dispensable for having phenomenally contrasting conscious states. In particular, sensory phenomenology seems dispensable when it comes to the phenomenology of conscious thought, so Kriegel says.

27 Note that liberalism* faces a challenge by a *strong* phenomenal holism. Chudnoff puts this view as follows: “Phenomenal Holism: *All* partial phenomenal states depend on the total phenomenal states they belong to.” (2015, p. 121). According to this strong version of phenomenal holism, cognitive phenomenal parts would depend on the overall experience which includes also sensory phenomenal parts. Hence, when we consider our *actual* experiences, cognitive phenomenology would not be independent from sensory phenomenology and liberalism would also face a challenge by WPH. (However, as Chudnoff notes (2015, p. 121f.), strong phenomenal holism is consistent with the *possibility* of cognitive phenomenal states in the absence of sensory phenomenal states.).

28 Opponents of this view are, e.g., Pautz (2013), Carruthers and Veilett (2011), Robinson (2011).

29 In what follows, I take concepts to be mental representations (Fodor 1998) rather than Fregean senses or abilities.

30 Nes’ account is compatible with the view that concepts make a constitutive contribution to proprietary cognitive phenomenology and also with the view that concepts make a constitutive contribution to sensory phenomenology.

31 As Nes argues (2011, p. 99f.), fusing and conjoining non-conceptual, sensory-phenomenal, parts does not suffice to explain objectual unity and Gestalt phenomena. Plausibly, fusion of these elements alone cannot account for the phenomenology of thought either. I agree with his diagnosis. However, while Nes concludes that thematic unity is key for the phenomenology of thought, I propose that the hybrid phenomenal state is key.

32 Fusion is compatible with both holism and atomism. One plausible picture is that the cognitive phenomenal part is not influenced by the overall state since it is the stable factor that can be combined with various sensory phenomenologies. Accordingly, WPH would apply only to the sensory phenomenal realm and need not extend to the cognitive phenomenal aspect.

33 This differentiates the fusion account from Levine’s (2011) notion of “impure cognitive phenomenology”. Impure cognitive phenomenology is a cognitively inflected sensory phenomenology. However, according to Levine, “the only way to phenomenally experience a cognitive content is through its effect on some sensory presentation.” (2011, p. 112). In contrast, on the fusion account, the phenomenology of conscious thought is a new hybrid phenomenology, over and above sensory phenomenology.

34 Schwitzgebel & Gordon (2000) argue that also sighted individuals have the capacity to echolocate to some extent but they do not notice this experience introspectively. Hence, it is unlikely that sighted individuals use imagery of echolocation to represent spatial distances in conscious thought.

35 Phenomenal functionalism assigns priority to the function and, hence, allows such high variation in the sensory basis as well. However, it explains the sameness of content via non-phenomenal functional facts and, hence, does not qualify as a strong CP-thesis.

36 The sensory phenomenology need not be visual imagery. For example, one can think *that thunderstorms are dangerous* without any visual imagery of thunderstorms or lightnings. The sensory phenomenology tied to this thought could also be an auditory imagery (an inner hearing of a thunder), etc.

37 As an anonymous referee points out, also conservatives might endorse the weaker claim that some (but no particular) sensory phenomenology is needed for having a conscious thought. However, such conservative view cannot explain how phenomenology secures determinate thought content. In contrast, the fusion account fits equally well with WPH *and* explains the constitution of thought content via phenomenology.

38 Thanks to an anonymous referee for drawing my attention to this line of thought.

39 For a discussion of the content-indeterminacy problem of sensory phenomenology, see Horgan and Graham (2012) and Pitt (2004).
